# Immunotherapy for Melanoma: The Significance of Immune Checkpoint Inhibitors for the Treatment of Advanced Melanoma

**DOI:** 10.3390/ijms232415720

**Published:** 2022-12-11

**Authors:** Taku Fujimura, Yusuke Muto, Yoshihide Asano

**Affiliations:** Department of Dermatology, Tohoku University Graduate School of Medicine, 1-1 Seiryo-machi, Aoba-ku, Sendai 980-8574, Japan

**Keywords:** immune checkpoint inhibitors, advanced melanoma, anti-PD1 Abs, anti-CTLA4 Abs, efficacy, irAEs

## Abstract

Therapeutic options for treating advanced melanoma have progressed rapidly in recent decades. Until 6 years ago, the regimen for treating advanced melanoma consisted mainly of cytotoxic agents such as dacarbazine and type I interferons. Since 2014, anti-programmed cell death 1 (PD1) antibodies have been recognized as anchor drugs for treating advanced melanoma, with or without additional combination drugs such as ipilimumab, but the efficacies of these immunotherapies are not fully satisfactory. In this review, we describe the development of the currently available anti-PD1 Abs-based immunotherapies for advanced melanoma, focusing on their efficacy and immune-related adverse events (AEs), as well as clinical trials still ongoing for the future treatment of advanced melanoma.

## 1. Introduction

In recent decades, immune checkpoint inhibitors (ICIs) and anti-programmed cell death 1 (PD1) antibodies (Abs) have been recognized as anchor drugs for the treatment of advanced melanoma, with or without additional drugs such as anti-CTLA-4 Abs in combination [1,2]. Since anti-PD1 Abs are widely applicable to the treatment of advanced melanoma even without *BRAF* mutations, anti-PD1 Ab-based regimes for the treatment of advanced melanoma have recently been developing [3,4,5]. This review describes the development of the currently available anti-PD1 Ab-based immunotherapies for advanced melanoma, focusing on their efficacy and immune-related adverse events (irAEs), as well as clinical trials still ongoing for the future treatment of advanced melanoma.

## 2. Significance of the Blockade of PD1/PD-L1 Pathways for the Treatment of Melanoma

### 2.1. Anti-PD-1Ab Monotherapy for Melanoma

ICIs are now among the first-line treatments for melanoma, and they are widely used to treat advanced melanoma even without BRAF mutations [3,4,5]. Anti-programmed cell death 1 antibodies (PD1 Abs) are in wide use for the treatment of various cancers [6,7], of which melanoma is the first cancer type for which their use has been approved by the US Food and Drug Administration (FDA). Since cutaneous melanoma, except for acral and mucosal melanoma, possesses a high tumor mutation burden (TMB) [8], anti-PD1 Ab monotherapy is given in both adjuvant [9,10,11] and unresectable settings [1,12,13].

#### 2.1.1. Anti-PD1 Ab Monotherapy for Unresectable Melanoma

Anti-PD1 Abs, nivolumab and pembrolizumab, are available for advanced melanoma treatment in both unresectable and adjuvant settings [5,6,7,8,9,10,11,12,13]. In the first trial to assess the efficacy of nivolumab for unresectable melanoma (Checkmate 066 trial), nivolumab (3 mg/kg) improved 1-year overall survival (OS) compared to dacarbazine (1000 mg/m^2^) in untreated advanced melanoma patients without BRAF mutations (72.9% vs. 42.1%) [14]. Since the estimated five-year overall survival (OS) rate was 44% and the 5-year progression-free survival (PFS) rate was 29% with nivolumab monotherapy for *BRAF*-wild-type advanced melanoma, which was comparable to those of nivolumab plus ipilimumab combination therapy [2], nivolumab monotherapy was considered a first-line immunotherapy for *BRAF*-wild-type advanced cutaneous melanoma [3,4,5].

Monotherapy with another anti-PD1 Ab, pembrolizumab, for unresectable melanoma should also be considered first-line immunotherapy for advanced cutaneous melanoma [13,15]. The objective response rate (ORR) of pembrolizumab was 36% for unresectable melanoma in the Caucasian population, whereas the ORR was 24.1% (95% CI 10.3–43.5%) for cutaneous melanoma in a Japanese population. Median OS and median PFS were 32.7 months (95% CI 24.5–41.6 months) and 8.4 months (95% CI 6.6–11.3 months), respectively [13]. The 5-year OS rate was 38.7% (95% CI 34.2–43.1%), and the 48-month PFS rate was 23.0% (95% CI 19.1–27.1%) in the pembrolizumab group [13].

#### 2.1.2. Anti-PD1 Ab Monotherapy for Melanoma in the Adjuvant Setting

Since melanoma is one of the most fatal skin tumors, several adjuvant therapies for advanced melanoma have been developed over the decades [5]. Of them, both nivolumab and pembrolizumab are approved for melanoma at high risk of recurrence, especially BRAF wild-type melanoma [9,16,17]. Indeed, nivolumab achieved a 4-year recurrence-free survival (RFS) rate of 51.7% and a 4-year OS of 77.9% in stage III-IV melanoma patients in the adjuvant setting [9]. Pembrolizumab also showed better 3.5-year distant metastasis-free survival than placebo treatment for resected stage III melanoma in the intention to treat population (65.3% [95% CI 60.9–69.5%] in the pembrolizumab group vs. 49.4% [44.8–53.8%] in the placebo group; HR 0.60 [95% CI 0.49–0.73]; *p* < 0.0001) [16]. In addition, pembrolizumab as adjuvant therapy for up to approximately 1 year for stage IIB or IIC melanoma resulted in a significant prolongation of RFS versus placebo [86% vs. 77%, hazard ratio (HR): 0.60 (95% CI: 0.45–0.79)], with an acceptable safety profile [18]. Moreover, pembrolizumab in the adjuvant setting for stage IIB or IIC melanoma significantly reduced the onset of distant metastasis versus placebo (6% vs. 12%) [18].

### 2.2. Combination Therapy with Anti-CTLA4 and Anti-PD-1 Inhibitors

#### 2.2.1. Nivolumab Plus Ipilimumab Combination Therapy for Unresectable Melanoma

Nivolumab plus ipilimumab combination therapy is recommended as a first-line immunotherapy for advanced melanoma [3,4]. Since the ORR to nivolumab plus ipilimumab combination therapy is higher than that for nivolumab monotherapy [57.6% (95% CI 52.0–63.2%) vs. 43.7% (95% CI 38.1–49.3%)] [1], this combination therapy is widely used for the treatment of advanced melanoma with or without BRAF mutations despite its high incidence of severe or serious adverse events (SAEs) [5]. Notably, in patients with acral melanoma (palm and sole melanoma) in the Japanese population, the efficacy of nivolumab plus ipilimumab combination therapy was not significantly better than that of anti-PD1 Ab monotherapy [19]. Indeed, the ORR was 31% (*p* = 0.44), the PFS was 3.2 months (*p* = 0.74) and the OS was not reached (*p* = 0.55) in the nivolumab plus ipilimumab groups [19]. On the other hand, in non-acral melanoma in the Japanese population, the ORR to nivolumab plus ipilimumab was significantly higher than that of anti-PD1 Ab monotherapy (61% vs. 10%; *p* < 0.001) in the same retrospective study [19]. Collectively, similar to anti-PD1 Abs [12], the efficacy of nivolumab plus ipilimumab combination therapy is determined, at least in part, by the clinical subtype of melanoma.

Several serological factors affect the efficacies of nivolumab plus ipilimumab combination therapy and anti-PD1 Ab monotherapy [2,20,21]. Of the routine blood tests, lactate dehydrogenase (LDH) could be one of the most relevant serological factors for the prediction of the clinical benefits of nivolumab plus ipilimumab combination therapy [2]. In patients with elevated LDH levels, the 5-year OS rate and PFS rate were much better with nivolumab plus ipilimumab combination therapy (38%, 28%) than with nivolumab monotherapy (28%, 18%) [2]. C reactive protein (CRP) and IL-6 are another serological factor for the prediction of the clinical benefits of nivolumab with or without ipilimumab therapy [22,23]. Indeed, higher levels of CRP and IL-6 are associated with prognostic factors with shorter OS in patients with metastatic melanoma receiving ICIs [22,23]. Notably, nivolumab plus ipilimumab combination therapy in the neoadjuvant setting dramatically expanded tumor-resident T cell clones that could directly eliminate the melanoma at primary tumor sites [24], suggesting the importance of ipilimumab for the treatment of advanced melanoma with elevated LDH levels and high tumor burden. Indeed, nivolumab plus ipilimumab combination therapy was effective for the late phase of advanced melanoma with 7 metastatic organs in a real-world setting [25]. Collectively, since ipilimumab expands tumor-resident and specific T cell clones at tumor sites, and since nivolumab abrogates the tolerance of these T cell clones against melanoma, elevated LDH levels (high tumor burden) could be relevant serological factors for the prediction of the clinical benefits from nivolumab plus ipilimumab combination therapy. *BRAF* mutations could be another predictor of the efficacy of nivolumab plus ipilimumab combination therapy.

Subgroup analysis of the CheckMate 067 trial showed the utility of nivolumab plus ipilimumab combination therapy for the *BRAF*-mutated advanced melanoma group [2]. Indeed, the 5-year OS and PFS rates were much better in the nivolumab plus ipilimumab combination group (60%, 38%) than in the nivolumab monotherapy group (46%, 22%) in patients with tumors with *BRAF* mutations [2]. Since the NCCN guideline for melanoma recommended the combination of BRAF plus MEK inhibitor, such as dabrafenib plus trametinib combination therapy, encorafenib plus binimetinib combination therapy and vemurafenib plus cobimetinib combination therapy, for the treatment of *BRAF*-mutated advanced melanoma [1], selecting the preferred initial treatment sequence in this population is of interest to dermato-oncologists [26]. A recent phase III study (doublet, randomized evaluation in advanced melanoma sequencing, DREAMseq; NCT02224781) showed that the treatment sequence beginning with nivolumab plus ipilimumab combination therapy provided better OS than the treatment sequence beginning with dabrafenib plus trametinib [27]. Another clinical trial also suggested that the efficacy of BRAF/MEK inhibitors for patients with elevated LDH levels was limited [28]. Collectively, nivolumab plus ipilimumab combination therapy could be a first-line immunotherapy for *BRAF*-mutated advanced melanoma with a high tumor burden.

#### 2.2.2. Nivolumab Plus Ipilimumab Combination Therapy for Melanoma with Brain Metastasis

The efficacy of nivolumab plus ipilimumab for melanoma with brain metastasis is still controversial because clinical trials generally exclude such patients [1,2,14]. The efficacy of nivolumab plus ipilimumab for melanoma with brain metastasis is as follows: the median OS achieved with nivolumab/ipilimumab combination therapy was unreached (median follow-up, 14 months); the 1-year survival rate for nivolumab plus ipilimumab combination therapy in patients with asymptomatic melanoma with brain metastasis without previous local therapy was 81.5% (95% CI, 71.5–88.2%) [29]. On the other hand, the results of a multicenter, open-label phase II trial that assessed the efficacy of dabrafenib plus trametinib combination therapy (COMBI-MB) for melanoma with brain metastasis were as follows: median OS was 10.8 months (95% CI, 8.7–19.6 months) for the BRAFV600E, asymptomatic brain metastasis with no prior local therapy group, 24.3 months (95% CI, 7.9–unreached months) in the BRAFV600E, asymptomatic brain metastasis with prior local therapy group, 10.1 months (95% CI, 4.6–17.6 months) in the BRAFV600D/K/R, asymptomatic brain metastasis with or without prior local therapy group, and 11.5 months (95% CI, 6.8–22.4 months) in the BRAFV600D/K/R, symptomatic brain metastasis, with or without prior local therapy group [30].

#### 2.2.3. Anti-PD1 Abs Monotherapy for Melanoma in the Neoadjuvant Setting

A recent clinical trial showed the effectiveness of nivolumab plus ipilimumab combination therapy in the neoadjuvant setting for the treatment of resectable melanoma with high risk, suggesting that this combination therapy achieved a high pathologic response rate (pRR: 74–78%) and prolonged RFS (94–100%) in responder patients at 2 years [27,31]. More recently, another clinical study (PRADO trial) confirmed the efficacy of this combination therapy [32], suggesting that the 24-month RFS and distant metastasis-free survival were 93% and 98%, respectively, in patients who achieved a major pathological response (≤10% viable tumor), 64% and 64%, respectively, in patients with a pathological partial response (PR), and 71% and 76%, respectively, in patients with a pathological non-response [32]. Although the neoadjuvant protocol using ipilimumab 3 mg kg^−1^ and nivolumab 1 mg kg^−1^ resulted in SAEs (G3/G4) at a high rate (90%) [24], the protocol using ipilimumab 1 mg kg^−1^ and nivolumab 3 mg kg^−1^ in the neoadjuvant setting was well-tolerated, with SAEs in 22% [32]. Notably, ipilimumab activates, increases, and expands effector T cells at the tumor site when given with nivolumab, leading to the effective induction of the anti-tumor immune response against melanoma [24]. Collectively, nivolumab plus ipilimumab in the neoadjuvant setting could be the optimal therapy for advanced melanoma with high risk.

### 2.3. Anti-CTLA4 Inhibitor: Ipilimumab Monotherapy

The cytotoxic T-lymphocyte-associated protein 4 (CTLA-4) inhibitor, ipilimumab, was the first immune checkpoint inhibitor approved by the FDA for use in advanced melanoma in 2011. The main mechanisms of ipilimumab inducing the anti-tumor immune response are the blockade of CTLA-4-enhanced T cell priming by tumor antigen presentation and the suppression of regulatory T cell (Treg) function, leading to activated effector T cell infiltration in the tumor microenvironment [33]. In addition, ipilimumab depletes Tregs to abrogate immune tolerance in tumor-bearing hosts [5,34]. According to previous clinical trials, ipilimumab (3 mg/kg) provided a longer median OS in unresectable stage III or IV melanoma patients than the gp100 peptide vaccine alone (NCT00094653/CA184–002) (10.1 months vs. 6.4 months) [35]. In another phase III clinical trial (NCT00324155), the combination of ipilimumab (10 mg/kg) plus dacarbazine (850 mg/m^2^ of body surface area) showed significantly better OS than dacarbazine (850 mg/m^2^) and placebo in untreated melanoma patients (11.2 months vs. 9.1 months) [36]. Since the efficacy of nivolumab monotherapy is superior to that of ipilimumab for the treatment of unresectable melanoma [1], ipilimumab monotherapy is not recommended as a first-line therapy for unresectable melanoma [3]. However, ipilimumab is still important for the treatment of advanced melanoma as a combination drug together with nivolumab.

### 2.4. Anti-PD-L1 Inhibitor: Atezolizumab Monotherapy and Combination Therapy

Similar to anti-PD1 Abs, anti-PD-L1 Abs are also recommended for the treatment of unresectable advanced melanoma in the USA, especially as a combination therapy with cobimetinib plus vemurafenib [37,38,39]. A randomized, double-blind, placebo-controlled phase 3 study (IMspire150) showed that the PFS of atezolizumab with vemurafenib plus cobimetinib combination therapy was significantly longer than that of vemurafenib plus cobimetinib combination therapy for the treatment of BRAFV600 mutation-positive unresectable melanoma (15.1 vs. 10.6 months; hazard ratio [HR] 0.78; 95% CI 0.63–0.97; *p* = 0.025) [37]. ORRs and profiles of irAEs were similar between these two groups [atezolizumab (66.3%; 95% CI 60.1–72.1%) and control groups (65.0%; 58.7–71.0%)] [37]. Moreover, another multicenter, open-label, single-arm, phase 2 study (TRICOTEL) showed that atezolizumab with vemurafenib plus cobimetinib combination therapy was even effective for BRAFV600 mutation-positive melanoma with brain metastasis [40]. The intracranial ORR was 42% (95% CI: 29–54%) for atezolizumab with vemurafenib plus cobimetinib combination therapy, which is comparable to that reported with other available systemic treatments described in Section 2.1.2 (ORRs of nivolumab plus ipilimumab 46%, dabrafenib plus trametinib 55% [29,30]. In addition to BRAFV600 mutation-positive unresectable melanoma, atezolizumab was also evaluated in combination therapy with cobimetinib for the treatment of BRAFV600 wild-type unresectable melanoma [38]. An international, randomized, open-label, phase III study reported that median PFS was 5.5 months (95% CI 3.8–7.2 months) with cobimetinib plus atezolizumab versus 5.7 months (95% CI 3.7–9.6 months) with pembrolizumab [HR 1.15 (95% CI 0.88–1.50); *p* = 0.30] [38]. The ORR was 26.0% (95% CI 20.1–32.6%) with cobimetinib plus atezolizumab versus 31.6% (95% CI: 25.3–38.4%) with pembrolizumab [38]. These results suggest that cobimetinib plus atezolizumab does not improve PFS compared with pembrolizumab monotherapy in patients with BRAFV600 wild-type advanced melanoma [38]. Another clinical study evaluated the efficacy of atezolizumab monotherapy as a first-line therapy for unresectable melanoma [39]. The ORR of atezolizumab monotherapy for BRAFV600 wild-type was 35% (95% CI, 22–49%), including three CRs (6%), and the DCR was 46%. The median investigator-assessed PFS was 3.7 months (95% CI, 2.1–7.3 months). Collectively, compared to other ICIs, atezolizumab combined with vemurafenib plus cobimetinib could be recommended for the treatment of BRAFV600 mutation-positive unresectable melanoma, even with CNS metastasis.

### 2.5. ICIs for Melanoma in Asian Population

The efficacy of anti-PD1 Ab monotherapy is limited in the Asian population [12]. The ORR of nivolumab monotherapy was 43.7% (95% CI 38.1–49.3%) in a Caucasian population [14], but lower in a Japanese population (34.8%; 95% CI 20.8–51.9%) [41]. In addition to clinical trials, post-marketing surveillance in Japan also showed that the ORR of nivolumab monotherapy was much lower in a Japanese population than in a Caucasian population (22.2%) [42]. This discrepancy could be explained by the high ratio of acral melanoma in the Japanese population (40.4%) [43]. Since the number of structural variants mutations is significantly lower in acral melanoma than in cutaneous melanoma [8], and since the efficacy of anti-PD1 Abs, at least in part, depends on TMB [8,44], the efficacy of anti-PD1 Abs for advanced acral melanoma should be limited [3]. Indeed, the ORR of anti-PD1 Abs is lower for unresectable acral melanoma (16.6%) [12] than for cutaneous melanoma (43.7%) [1], and the PFS and OS for the study cohort were 3.5 months and 18.2 months, respectively [12]. Moreover, another report suggested that, although Cox multivariate analysis suggested that performance states (PS) and elevated LDH is an independent predictor of a favorable OS and PS in the palm and sole melanoma, the efficacy of nivolumab plus ipilimumab combined therapy is not superior to that of anti-PD1 Abs for the treatment of advanced acral melanoma [19]. Surprisingly, this tendency is observed not only in the Asian population but also in other ethnic populations, including Hispanic and African patients with melanoma [45]. In addition, the efficacy of anti-PD1 Abs as adjuvant therapy for up to approximately 1 year for stage III melanoma was significantly lower in acral melanoma than in cutaneous melanoma in a Japanese cohort (31 acral melanoma vs. 31 cutaneous melanoma patients, *p* = 0.026) [9]. The safety profiles of nivolumab and pembrolizumab in the adjuvant setting are comparable to those for unresectable melanoma [9,16,17]. Collectively, anti-PD1 Abs in the adjuvant setting are less effective for acral melanoma than for cutaneous melanoma. Therefore, it will be important to evaluate the differences in the effect of anti-PD1 Abs in the adjuvant setting between populations of different racial backgrounds in the future. Since the efficacy of anti-PD1 Ab monotherapy is limited in the ethnic populations including an Asian population [12,45], the development of anti-PD1 Ab-based combination therapy or biomarkers for the prediction of the efficacy of anti-PD1 Abs are needed in the future.

## 3. Adverse Events of ICIs

### 3.1. Skin Reactions as irAEs

As described above, ICIs such as anti-PD1 Abs and anti-CTLA4 Abs have become anchor drugs in the treatment of advanced melanoma [1,2,5] (Table 1). ICIs significantly prolong survival in patients with metastatic melanoma [1,2,13], but the associated risk of irAEs is an important consideration [46,47,48]. Among such irAEs, dermatological toxicities are among the most common, well-known and earliest onset irAEs [46,47,48]; however, especially in clinical studies, dermatological toxicities are categorized as “skin eruption”, and they were not further described. Since cutaneous irAEs could present with various manifestations, and since the treatment for these cutaneous irAEs is different for each phenotype [46,47], the classification of cutaneous irAEs is important for the safe use of ICIs. According to previous reports [46,47,48], cutaneous irAEs are roughly classified as follows: (1) enhancement of other inflammatory skin diseases (e.g., drug eruption, psoriasis, lichen planus); (2) induction of substantial autoimmune skin disease (e.g., bullous pemphigoid); (3) melanocytic skin reaction (e.g., vitiligo).

#### 3.1.1. Enhancement of Skin Inflammatory Disease

The most common subtypes of skin toxicity caused by ICIs are maculopapular rashes and a lichen planus-like lichenoid skin reaction [46,47]. The incidence rate of maculopapular rashes after anti-PD-1 Abs treatment is 24%, which may include pre-symptoms of other skin inflammatory irAEs [49]. Since ICIs enhance not only anti-tumor immune responses but also other immune reactions, including skin reactions, hypersensitivity reactions against other drugs, including Stevens-Johnson syndrome/toxic epidermal necrolysis (Sjs/TEN), drug reaction with eosinophilia and systemic symptoms (DRESS), and acute generalized exanthematous pustulosis (AGEP), could develop during ICI administration [49,50,54,64]. Lichenoid reactions are prominent, especially in patients treated with anti-PD-1/PD-L1 agents as early-onset cutaneous irAEs [51]. The mean time to onset of lichenoid dermatological toxicity was 42 days (range: 1–75 days) from the initiation of anti-PD-1 antibody therapy [51].

Since these cutaneous irAEs described above suggest enhancement of the immune responses in tumor-bearing hosts, the onset of irAEs might correlate with the efficacy of ICIs for the treatment of advanced melanoma [53,62,63]. Indeed, the appearance of any cutaneous irAEs was significantly protective against mortality (hazard ratio (HR): 0.778; 95% CI 0.725–0.834; *p* < 0.001) [53]. Moreover, several reports focused on vitiligo as a cutaneous irAE that was correlated with a good prognosis of advanced melanoma treated with anti-PD1 Abs [62,63]. For example, vitiligo induced by anti-PD1 Abs is a factor associated with a good prognosis in patients with melanoma, and melanocyte/melanoma-shared antigen (MSA)-specific CD8+ T cells play significant roles in the prognosis of melanoma [62]. In another report, both the onset of vitiligo and an increased serum CCL19 level were correlated with a better prognosis in advanced melanoma patients treated with anti-PD1 Abs [63]. Collectively, cutaneous AEs might be a manifestation that predicts a good response to ICIs such as anti-PD1 Abs [52].

#### 3.1.2. Common Cutaneous Disease Induced by ICIs as Cutaneous irAEs: Bullous Pemphigoid, Lichen Planus, Psoriasis

Bullous pemphigoid is one of the common cutaneous AEs that occurs in 1% of all patients treated with anti-PD-1/PD-L1 Abs [55]. A recent report also suggested that the levels of anti-BP180 IgG were correlated with ORR and OS, as well as a propensity to develop skin irAEs during anti-PD-1/PD-L1 treatment in patients with non-small cell lung cancer [56]. Histologically, CD163+ M2 macrophages are prominent in the lesional skin of bullous pemphigoid [65], and soluble (s)CD163 is increased in serum from patients with bullous pemphigoid compared to healthy donors [66]. Notably, CD163+ tumor-associated macrophages (TAMs) in melanoma express both PD1 and PD-L1, and blockade of PD-L1/PD1 signals by anti-PD1 Abs activates TAMs [67,68], leading to the production of TAM-activating factors such as sCD163 and chemokines in the serum of melanoma patients [57]. In addition, serum levels of sCD163 were significantly correlated with the efficacy of anti-PD1 Abs [57]. Thus, bullous pemphigoid-like cutaneous irAEs caused by anti-PD1/PD-L1 Abs might correlate with the prognosis of advanced melanoma.

Lichen planus is also a well-known cutaneous irAE [52,57]. Similar to conventional lichen planus, dermal-infiltrating lymphocytes in lichen planus induced by ICIs express phosphor-signal transducers and activators of transcription (pSTAT)1 [58,69], suggesting that ICIs could cause a Th1-polarized systemic anti-tumor immune response [70]. Indeed, the group that developed cutaneous irAEs, including lichen planus, had significantly prolonged OS compared with the group that did not develop cutaneous irAEs in patients treated with anti-PD1 Abs [52], suggesting that lichen planus could be a cutaneous manifestation that predicts a good prognosis in melanoma patients treated with anti-PD1 Abs. Lichen planus pemphigoides is a rare cutaneous manifestation that could develop from lichen planus lesions induced by various factors, including by ICIs such as nivolumab, pembrolizumab and atezolizumab [59,60,61]. Since the number of reported cases is limited, the correlation between prognosis and lichen planus pemphigoides-like irAEs is still unknown.

### 3.2. Systemic irAEs Caused by ICIs

Since ICIs could enhance the systemic immune system, various organs could be a target of irAEs that present as autoimmune diseases [64]. Pneumonia, hepatitis, colitis and endocrine disorders (hypothyroidism, hypophysitis, diabetes mellitus, etc.) [71,72] are common irAE caused by the blockade of PD1/PD-L1 pathways and/or CTLA4 (Table 1) [61]. Although the guidelines for the management of these irAEs described above are well established [61], severe irAEs of these organs, as well as minor irAEs such as neurotoxicity [73,74], ocular disorders [75,76], muscle skeletal disorders [77,78] and cardiovascular disorders [79], are still major problems with the use of ICIs. Importantly, the onset of these irAEs was correlated with increased serum levels of CXCL5 [72,78,80], a well-known biomarker for the activity of autoimmune disorders such as rheumatoid arthritis [81,82]. Since serum levels of proinflammatory chemokines such as CXCL5 could predict not only the onset of irAEs but also the efficacy of ICIs [65,83], several irAEs might correlate with the efficacy of ICIs [84,85,86]. Indeed, a retrospective analysis of 190 melanoma patients treated with anti-PD1 Abs in the USA showed that irAE occurrence was significantly associated with the efficacy (ORR, improved OS and PFS) of anti-PD1 Abs monotherapy, especially associated with cutaneous and arthritis-like irAEs [84]. Another report also suggested an association of irAEs with efficacy (ORR, improved OS and PFS) of any type of ICIs, but SAEs resulted in better ORR, but worse OS [85].

## 4. Other ICI-Based Combination Therapies

### 4.1. Combination Therapy with LAG-3–Blocking Antibody and Nivolumab

As described above, although anti-PD1 Abs in combination with ipilimumab significantly prolonged survival in patients with metastatic melanoma, the high frequency of irAEs needs to be addressed [1,2]. Therefore, several clinical trials have been designed to enhance the efficacy of anti-PD1 Abs. Among them, an anti-lymphocyte activation gene-3 (LAG-3) Ab, relatlimab, is another ICI that could be combined with nivolumab for the treatment of untreated advanced melanoma [87]. Indeed, relatlimab plus nivolumab combination therapy for untreated advanced melanoma was evaluated in a phase 2–3, global, double-blind, randomized trial [87], suggesting that the PFS of relatlimab plus nivolumab combination therapy was significantly prolonged compared with nivolumab monotherapy (10.1 months (95% CI: 6.4–15.7 months) vs. 4.6 months (95% CI: 3.4–5.6 months) (HR: 0.75 [95% CI: 0.62–0.92]; *p* = 0.006) [87]. PFS at 12 months was 47.7% (95% CI: 41.8%- 53.2%) for this combination therapy and 36.0% (95% CI: 30.5–41.6%) for nivolumab monotherapy [87]. Notably, the incidence of treatment-related SAEs was 18.9% in the relatlimab plus nivolumab combination therapy group and 9.7% in the nivolumab monotherapy group, suggesting the safety and tolerability of this combination therapy [87]. More recently, a clinical trial of relatlimab plus nivolumab combined therapy in a neoadjuvant setting evaluated the treatment of resectable melanoma [88]. Patients were sequentially administered two neoadjuvant doses, surgical resection and 10 doses of adjuvant combination therapy [88]. The pathological (p)CR for this regimen was 57%, and the overall pathological response rate (OpRR) was 70% in 30 patients [88]. No grade3–4 irAEs were observed in the neoadjuvant setting. The 1- and 2-year RFS rates were 100% and 92%, respectively, for patients with any pathologic response [88]. Collectively, relatlimab plus nivolumab combination therapy in the neoadjuvant setting could provide a high pCR for the treatment of resectable melanoma.

### 4.2. Combination Therapy with Plasminogen Activating Inhibitor-1 (PAI-1) Inhibitors and Nivolumab

PAI-1 is a serine protease that correlates with a poor prognosis for various cancers, including melanomas [89,90]. Since PAI-1 facilitates PD-L1 endocytosis of melanoma cells to abrogate the efficacy of anti-PD-L1 Abs in mouse melanoma models [91], and since baseline serum PAI-1 levels and PAI-1 expression on melanoma correlate significantly with the efficacy of anti-PD1 Abs for the treatment of advanced melanoma [92], blockade of the PAI-1 signal in combination with anti-PD1 Abs might enhance the anti-tumor immune responses against melanoma growth. To address these unmet medical needs, especially in Japanese patients with advanced melanoma, a single-arm, phase 2 clinical trial to evaluate the efficacy and safety of nivolumab plus a PAI-1 inhibitor, TM5614, in Japanese patients with metastatic melanoma is ongoing (jRCT2021210029) [93].

## 5. Future Perspectives

As described above, ICIs are currently among the most promising therapies to induce long-acting anti-tumor effects, even in BRAFV600 mutated melanomas [1,2,3,4,26,94]. Indeed, recent phase II/III clinical studies of the treatment of patients with BRAFV600-mutated advanced melanoma suggested that ICIs are promising for the induction of long-term, anti-melanoma effects [26,94]. The efficacy of nivolumab plus ipilimumab combination therapy is superior to that of dabrafenib plus trametinib combination therapy for the treatment of mutated, treatment-naïve, advanced melanoma [26]. The 2-year OS for those starting on nivolumab plus ipilimumab was 71.8% (95% CI, 62.5–79.1%) and for those starting on dabrafenib plus trametinib, it was 51.5% (95% CI, 41.7–60.4%; log-rank *p* = 0.010). In addition, PFS tended to be longer in the nivolumab plus ipilimumab arm (*p* = 0.054) [26].

Another phase II clinical trial also suggested that the preferred sequence of systemic therapy was nivolumab plus ipilimumab combination therapy and enforafenib plus binimetinib combination therapy for the treatment of BRAFV600-mutated advanced melanoma (SECOMBIT) [94]. The treatment arm of this study was as follows: arm A received encorafenib plus binimetinib until progressive disease (PD), followed by ipilimumab plus nivolumab until the second PD; Arm B received ipilimumab plus nivolumab until PD followed by encorafenib plus binimetinib until the second PD; Arm C (sandwich or induction/maintenance) received encorafenib plus binimetinib for 8 weeks followed by ipilimumab plus nivolumab until PD followed by encorafenib plus binimetinib until the second PD. The results of this phase II clinical trial showed that the 2-year and 3-year OS rates were 65% (95% CI, 54–76%) and 54% (95% CI, 41–67%), respectively, in arm A, 73% (95% CI, 62–84%) and 62% (95% CI, 48–76%), respectively, in arm B, and 69% (95% CI, 59–80%) and 60% (95% CI, 58–72%), respectively, in arm C, suggesting that nivolumab plus ipilimumab combination therapy was the best first-line therapy for the induction of long-term, anti-melanoma effects [94]. Since the efficacies of ICIs for the treatment of advanced melanoma differ in patients of different racial backgrounds, as described above [4,9], the results of these clinical trials should be confirmed in real-world settings in each country in the future.

## Figures and Tables

**Table 1:** Series of cutaneous AEs.

**Enhance skin inflammation**	**References**
Maculopapular rashes	[49,50]
Lichen planus-like lichenoid skin reaction	[49,50,51]
Urticaria	[50,52]
Neutrophilic dermatitis	[50]
Eczematous dermatitis	[50,52,53]
Erythema multiforme/Stevens–Johnson syndrome	[54,52]
**Common cutaneous disease induced by ICI**	**References**
Bullous pemphigoid	[52,53,55,56]
Lichen planus	[52,53,57,58]
Lichen planus pemphigoides	[59,60,61]
Psoriasis vulgaris	[49,50,52,53]
Vitiligo-like lesions	[49,53,62,63]
Dermatomyositis	[49]
Alopecia areata	[49]

## Data Availability

Not applicable.
